# Deep learning-based automatic-bone-destruction-evaluation system using contextual information from other joints

**DOI:** 10.1186/s13075-022-02914-7

**Published:** 2022-10-03

**Authors:** Kazuki Miyama, Ryoma Bise, Satoshi Ikemura, Kazuhiro Kai, Masaya Kanahori, Shinkichi Arisumi, Taisuke Uchida, Yasuharu Nakashima, Seiichi Uchida

**Affiliations:** 1 Department of Orthopaedic Surgery, Graduate School of Medical Sciences, Kyushu University, 3-1-1 Maidashi, Higashi-Ku, Fukuoka, 812-8582 Japan; 2 Department of Advanced Information Technology, Kyushu University, 744 Motooka, Nishi-Ku, Fukuoka, 819-0395 Japan

**Keywords:** Rheumatoid arthritis, Deep neural networks, Modified Sharp/van der Heijde score, Automatic detection, Automatic classification

## Abstract

**Background:**

X-ray images are commonly used to assess the bone destruction of rheumatoid arthritis. The purpose of this study is to propose an automatic-bone-destruction-evaluation system fully utilizing deep neural networks (DNN). This system detects all target joints of the modified Sharp/van der Heijde score (SHS) from a hand X-ray image. It then classifies every target joint as intact (SHS = 0) or non-intact (SHS ≥ 1).

**Methods:**

We used 226 hand X-ray images of 40 rheumatoid arthritis patients. As for detection, we used a DNN model called DeepLabCut. As for classification, we built four classification models that classify the detected joint as intact or non-intact. The first model classifies each joint independently, whereas the second model does it while comparing the same contralateral joint. The third model compares the same joint group (e.g., the proximal interphalangeal joints) of one hand and the fourth model compares the same joint group of both hands. We evaluated DeepLabCut’s detection performance and classification models’ performances. The classification models’ performances were compared to three orthopedic surgeons.

**Results:**

Detection rates for all the target joints were 98.0% and 97.3% for erosion and joint space narrowing (JSN). Among the four classification models, the model that compares the same contralateral joint showed the best F-measure (0.70, 0.81) and area under the curve of the precision-recall curve (PR-AUC) (0.73, 0.85) regarding erosion and JSN. As for erosion, the F-measure and PR-AUC of this model were better than the best of the orthopedic surgeons.

**Conclusions:**

The proposed system was useful. All the target joints were detected with high accuracy. The classification model that compared the same contralateral joint showed better performance than the orthopedic surgeons regarding erosion.

**Supplementary Information:**

The online version contains supplementary material available at 10.1186/s13075-022-02914-7.

## Introduction

Assessing the presence of bone destruction is important for diagnosing rheumatoid arthritis (RA) [1, 2]. In clinical settings, bone destruction is usually estimated as accurately as possible by observing X-ray images [3]. If the X-ray images reveal signs of bone destruction, the chance of an RA diagnosis is significantly increased, and early drug treatment is more likely to be suggested [4].

The modified Sharp/van der Heijde score (SHS) [5] is a commonly used metric for evaluating bone destruction by X-rays [6–8]. SHS has two assessment items: erosion and joint space narrowing (JSN). Erosion is assessed in 16 joints and JSN is assessed in 15 joints for each hand and wrist. SHS for erosion has six grades from 0 to 5, and SHS for JSN has five grades from 0 to 4 [5].

Among the grades, the classification between 0 (intact) and the others (non-intact) is the most important task for early diagnosis of RA [6]. However, performing this binary classification by visual inspection is difficult, even for RA experts, for three reasons.

First, binary classification of all joints by visual inspection is time-consuming [1, 9–11]. Second, accurate and stable classification requires extensive practical experience. Third, although each RA expert attempts the classification to the best of their ability, it is hard to avoid intra- and inter-expert variability. These three problems make it difficult to perform binary classification in actual clinical practice.

The purpose of this study is to propose a system for automatically evaluating bone destruction (hereafter, “automatic-bone-destruction-evaluation system”) by fully utilizing recent artificial intelligence (AI) techniques, called deep neural networks (DNN). The system first detects all target joints from a single X-ray image of the hands. It then classifies every target joint as “intact” (SHS = 0) or “non-intact” (SHS ≥ 1).

The contributions of this study are twofold. First, the proposed system is the first that can detect all the target joints automatically. As for methods developed in previous studies [12–14], the change in brightness is used to detect the target joints. Due to their complex structure, the target joints were limited to proximal interphalangeal (PIP), interphalangeal joint of the thumb (IP), and metacarpophalangeal (MCP) joints [12–14]. To detect all the target joints, we introduced a DNN model called *DeepLabCut*, which can detect various objects accurately by re-training the model with different targets [15].

Second, the proposed system is the first that performs binary classification for each joint while utilizing “contextual” information from other joints. As for RA, bone destruction progresses bilaterally and symmetrically [1, 3, 16–18]. Therefore, comparing both hands is useful when reading X-ray images of RA patients [9, 19–21]. Furthermore, the bone destruction of the same joint group, such as the PIP joints, tends to progress similarly [22, 23]. These comparisons of the related joints are often used in diagnosing RA. Therefore, we propose a method that evaluates the target joints using information concerning the relevant joints.

## Materials and methods

### Dataset

**Fig. 1:**
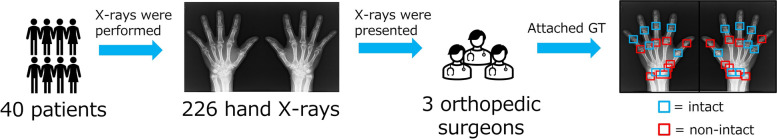
The flow of how the dataset was generated. The subjects of this study were 40 rheumatoid arthritis patients treated from 2008 to 2019 in 9 hospitals. All patients have both hands X-rayed. X-rays were taken at intervals of at least 1 year when more than one radiograph was taken for a single patient; 3 patients were X-rayed once, 5 patients were X-rayed twice, 30 patients were X-rayed 3 times, 1 patient was X-rayed 4 times, and 1 patient was X-rayed 6 times. In total, there were 113 both hands X-rays (i.e., 226 one-hand X-rays). Three orthopedic surgeons were presented with full hand X-rays and attached the ground-truth (GT) for each target joint as “intact” or “non-intact” by consensus

The Institutional Review Board approved this study. We used 226 hand X-ray images from 40 patients diagnosed with RA and treated with medication from 2008 to 2019 in nine hospitals (Fig. 1). Table 1 shows patients’ characteristics and breakdowns between hospitals. All X-rays contained the target joints completely. There were no images that were excluded by the image quality. Three orthopedic surgeons who treat RA attached the ground-truth (GT) for each target joint as “intact” or “non-intact” by consensus (Fig. 1). The numbers of intact and non-intact GTs for each joint are given in Table 2. Some joints are biased to be intact or non-intact, and this bias, called “class imbalance,” adversely affects the prediction performance of the DNN [24]. We applied the data augmentation [25] described below to mitigate this class imbalance.

**Table 1:** Patients’ characteristics for each hospital

	Site 1 ***n*** = 3	Site 2 ***n*** = 1	Site 3 ***n*** = 2	Site 4 ***n*** = 14	Site 5 ***n*** = 2	Site 6 ***n*** = 2	Site 7 ***n*** = 1	Site 8 ***n*** = 1	Site 9 ***n*** = 14	All ***n*** = 40
Age (years)	61.3 ± 16.1	71.0 ± 0.0	63.0 ± 0.0	60.0±10.1	73.5 ± 4.5	57.0 ± 9.0	73.0 ± 0.0	70.0 ± 0.0	59.5 ± 12.6	61.5 ± 11.6
Sex (male: female)	0: 3	0: 1	0: 2	2: 12	0: 2	0: 2	0: 1	0: 1	2: 12	4: 36
RA duration (years)	10.7 ± 9.5	N/A	14.0 ± 11.0	6.5 ± 4.6	20.0 ± 19.0	9.5 ± 1.5	13.0 ± 0.0	9.0 ± 0.0	15.1 ± 10.0	11.4 ± 9.7
MTX	3 (100%)	1 (100%)	2 (100%)	12 (86%)	1 (50%)	1 (50%)	1 (100%)	1 (100%)	11 (79%)	33 (83%)
Glucocorticoids	1 (33%)	1 (100%)	1 (50%)	13 (93%)	2 (100%)	2 (100%)	1 (100%)	0 (0%)	5 (36%)	26 (66%)
bDMARDs	2 (67%)	0 (0%)	1 (50%)	6 (43%)	0 (0%)	0 (0%)	1 (100%)	1 (100%)	9 (64%)	20 (50%)

Patients’ characteristics for each hospital are shown. Age and rheumatoid arthritis (RA) data represent average ± SD

*RA* rheumatoid arthritis, *MTX* methotrexate, *bDMARDs* biologic disease-modifying anti-rheumatic drugs

**Table 2:** Breakdown of the number of intact or non-intact images for each target joint

	Intact	Non-intact	Total
**Erosion**			
PIP-IP	1005	125	1130
MCP	987	143	1130
CMC-M	321	131	452
Wrist	434	470	904
All joints	2747	869	3616
**JSN**			
PIP	562	342	904
MCP	876	254	1130
CMC	328	350	678
Wrist	217	461	678
All joints	1983	1407	3390

The number of intact or non-intact images for each target joint is shown. For erosion, “Wrist” represents the navicular, the lunate, the radius, and the ulna; for JSN, “Wrist” represents the multangular-navicular, the capitate-navicular-lunate, and the radiocarpal joint

*PIP* proximal interphalangeal, *IP* interphalangeal, *MCP* metacarpophalangeal, *CMC-M* carpometacarpal joint of the thumb and multangular, *JSN* joint space narrowing, *CMC* carpometacarpal

### Method (overview)

**Fig. 2:**
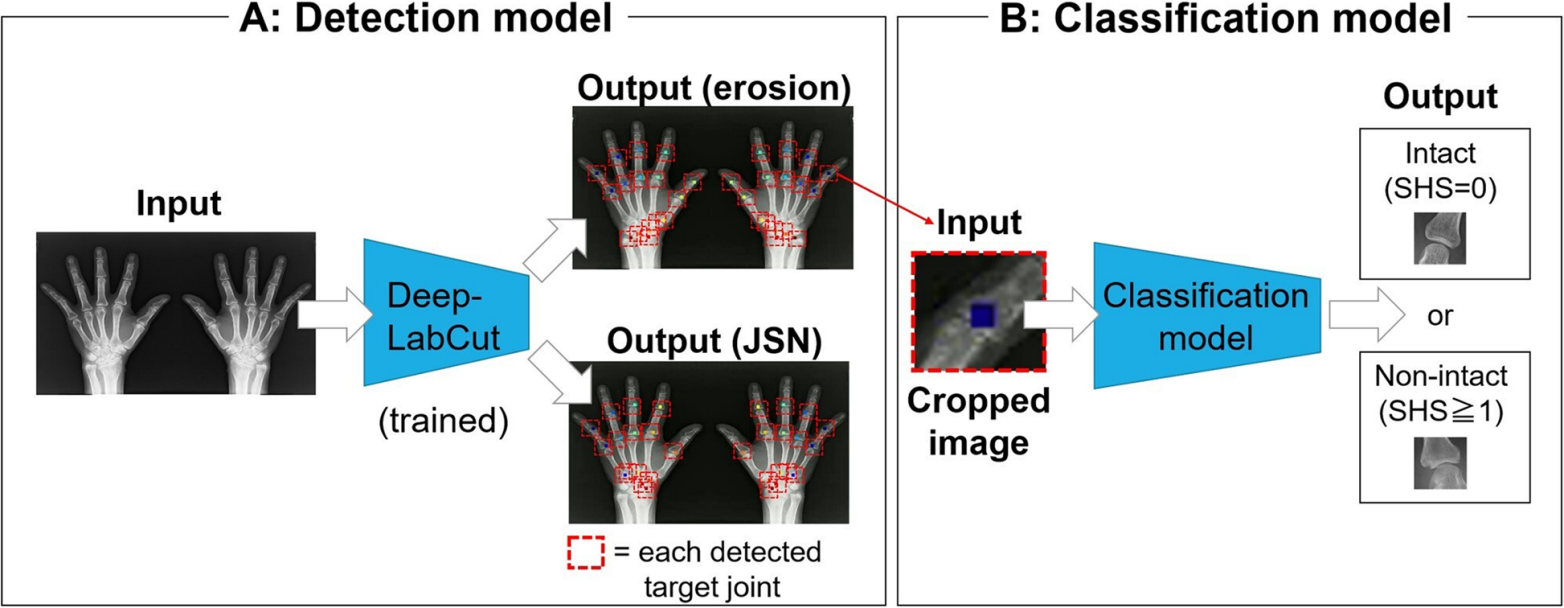
Overview of the proposed automatic-bone-destruction-evaluation system. **A** Input an X-ray image of hands into the detection model (DeepLabCut). DeepLabCut detects the center point of the evaluation joints of the SHS (target joints): 16 joints for erosion (the 4 PIP joints, the IP joint of the thumb, the 5 MCP joints, the CMC joint of the thumb, the multangular, the navicular, the lunate, the radius, and the ulna) and 15 joints for JSN (the 4 PIP joints, the 5 MCP joints, the 3 CMC joints, the multangular-navicular joint, the capitate-navicular-lunate joint, and the radiocarpal joint). Each point indicates the detected center of the target joints. Each joint image was cropped (red bounding box) according to the detected center point. **B** Each cropped image was input into the classification model, which outputs whether the input image is intact (SHS = 0) or non-intact (SHS ≥ 1). SHS, Sharp/van der Heijde score; PIP, proximal interphalangeal; IP, interphalangeal; MCP, metacarpophalangeal; CMC, carpometacarpal; JSN, joint space narrowing

Figure 2 overviews the proposed automatic-bone-destruction-evaluation system, which consists of three steps. First, a detection model detects the center point of the target joints (16 joints for erosion and 15 joints for JSN) from an inputted hand X-ray image (Fig. 2A). Next, each joint image is cropped around the detected center point. The cropped image is then input into the classification model for binary classification (Fig. 2B).

### Training of detection model

As the detection model, DeepLabCut [15], which was proposed for detecting and tracking an animal’s joints in video images, was used. DeepLabCut estimates each key point (joint) position from an input image. This detection model has three advantages: first, it can be applied to various objects by re-training it with a different target; second, it can be trained with a few labeled training data; and third, it can detect joints accurately by learning the structure of the joints (e.g., mutual positional relationships). We thus used DeepLabCut for detecting the target joints.

In the training of DeepLabCut, 20 X-ray images were randomly selected from 226 X-rays and resized to 256 × 256 pixels. Then, for each of the 20 images, the center points of the target joints were annotated by an orthopedic surgeon. Finally, two DeepLabCut models were trained with those training images: one to detect the target joints for erosion (16 joints) and the other to detect the target joints for JSN (15 joints).

### Models for classification

**Fig. 3:**
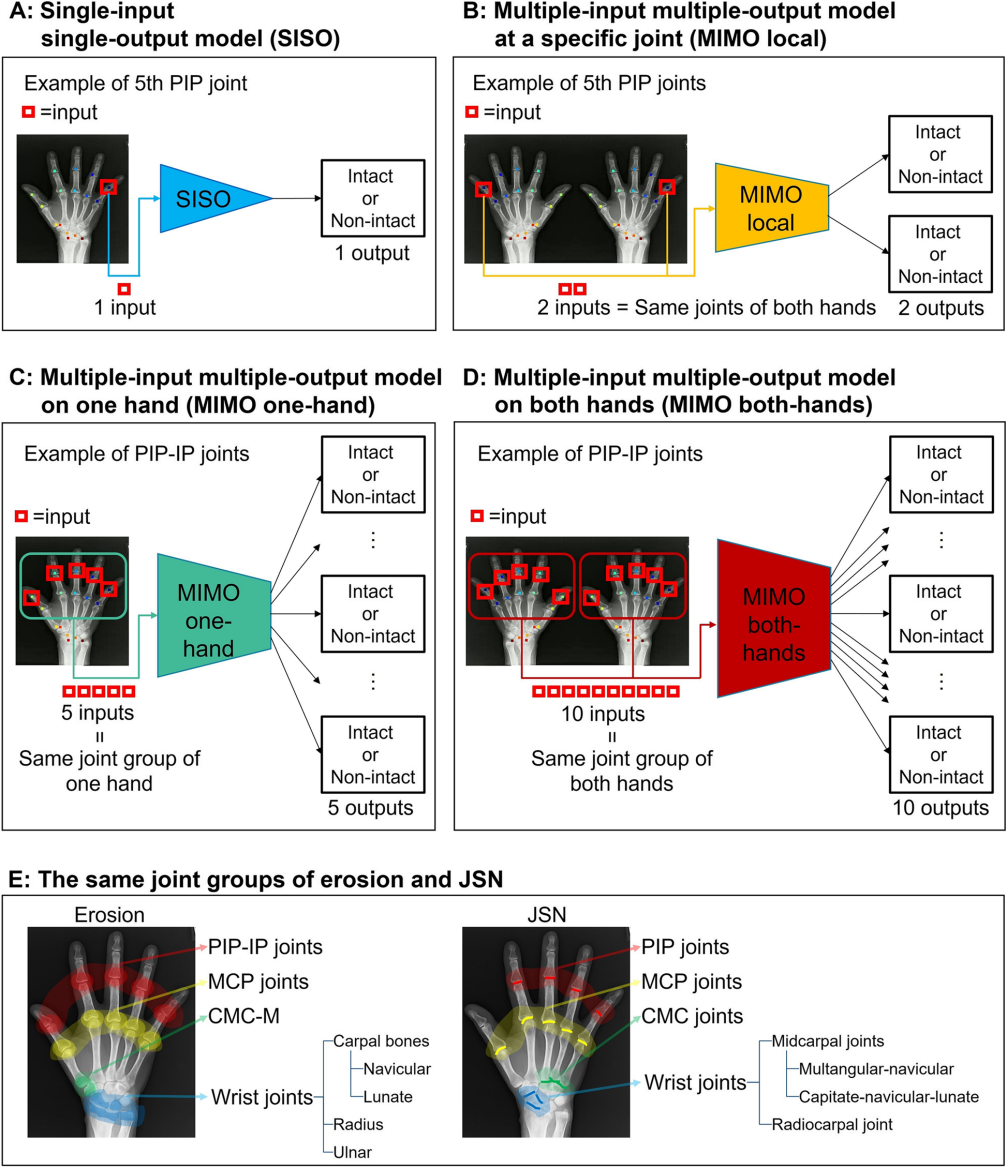
Overview of the binary classification model and the same joint groups. We developed four classification models: **A** A most-basic classification model that independently classifies each joint as intact (SHS = 0) or non-intact (SHS ≥ 1) (single-input single-output model). **B** A classification model that inputs the same joints of both hands and outputs intact or non-intact, respectively (multiple-input multiple-output model at a specific joint). **C** A classification model that receives inputs of the same joint group of one hand and outputs whether they are intact or non-intact, respectively (multiple-input multiple-output model on the one hand). **D** A classification model that receives inputs of the same joint group of both hands and outputs, whether intact or non-intact, respectively (multiple-input multiple-output model on both hands). **E** The same joint groups of both hands for erosion and JSN. PIP, proximal interphalangeal; IP, interphalangeal; MCP, metacarpophalangeal; CMC-M, carpometacarpal joint of the thumb and multangular; CMC, carpometacarpal

Several binary classification models were established, and their performances were compared via experiments. Figure 3A shows the baseline model, called the single-input single-output (SISO) model. The SISO model is the simplest and classifies each detected joint independently. In other words, it does not utilize information concerning other joints. As the backbone of the SISO model, a VGG16 [26], which has a typical convolutional neural network [27] structure and high object-recognition performance, was used. It is well-known that pre-training a neural network model with a large but non-target dataset boosts its performance. The VGG16 was therefore pre-trained by ImageNet [28] and then fine-tuned [29] by using (a limited number of) joint images.

In addition to the SISO model, three types of multiple-input multiple-output (MIMO) models were established. As discussed in the introduction, it is effective to compare the same contralateral joint [9, 19–21] or the same joint group [22]. From this viewpoint, the SISO model has room for improvement because it does not use the information on the relevant joints. The established MIMO models utilize information about the same contralateral joint and joint group (Fig. 3B–D).

For designing the MIMO models, the same joint group in one hand is defined as follows (Fig. 3E): for erosion, (1) the PIP and IP joints (PIP-IP joints), (2) the MCP joints, (3) the carpometacarpal (CMC) joint of the thumb and multangular (CMC-M), and (4) the wrist joints (the navicular, the lunate, the radius, and the ulna) [22, 23], and for JSN, (1) the PIP joints, (2) the MCP joints, (3) the CMC joints, and (4) the wrist joints (the multangular-navicular joint, the capitate-navicular-lunate joint, and the radiocarpal joint) [22, 23]. Given a set of joint images (the same joints of both hands or the same joint group) as inputs, the model simultaneously estimates the classes (“intact” or “non-intact”) of these multiple joints.

Figure 3B shows the *MIMO local* model, which can compare the same contralateral joint. This MIMO model receives inputs from the same joints of both hands and outputs “intact” or “non-intact” for each joint. Since the same (local) joints of both hands are compared, this model is referred to as the “MIMO local model.”

Figure 3C shows the *MIMO one-hand* model, which can compare multiple joints in the same joint group of one hand. This model gives all classification results for individual joints in their group using the mutual relationship.

Figure 3D shows the *MIMO both-hands* model, which can compare the same joint group of both hands. This model may take advantage of both the MIMO local model and the MIMO one-hand model.

### Training of classification models

The four classification models (SISO and three MIMOs) were trained under the following conditions. First, forty patients were divided into eight patient-disjoint subsets (i.e., five patients in each subset). This patient-disjointness is necessary to keep the fairness of our experiment [30]. Then, eightfold cross-validation was performed, where six subsets were used for training, one subset was used for validation, and one subset was used for testing. The classification models were trained by using the training images. The validation data was used to adjust hyperparameters and determine when to stop the training prematurely, namely, “early stopping,” which is used to improve the generalization of the test data [31].

The hyperparameters were as follows; the number of fully connected layers [32], the initialization scheme for fully connected layers (random or He normal initialization [33]), dropout [34], and batch size [35]. The hyperparameters were tuned to minimize the loss of validation data, and the final settings are described in Tables S1 and S2 in additional materials. The condition for early stopping was that validation loss does not decrease 10 times in a row. The training is terminated if this early stopping condition is not satisfied for 100 epochs. We set the binary cross-entropy loss [36] for the SISO model and the sum of the binary cross-entropy losses over all outputs for the MIMO models. The Adaptive Momentum (Adam) [37] was used as the optimizer for all models. Since image features among different joint groups differ significantly, different models were prepared for each group, as shown in Tables S1 and S2 in additional materials.

To address the class imbalance, we applied data augmentation, which improves the performance of the DNN when there is a class imbalance or only a small amount of training data [25]. We applied data augmentation to the training and validation data (for each joint of each fold in the cross-validation). Specifically, we applied -3- to 3-degrees rotations, -5- to 5-pixels vertical and horizontal translations, and 0.97- to 1.03-times enlargement (or reductions) to each cropped joint image. The geometric perturbations augmented the training data until the total number of images was about 10,000 with no class imbalance. The validation data were also augmented to remove the class imbalance.

### Procedure for evaluation of detection model

The performance of DeepLabCut was evaluated in the following two metrics using 206 test X-ray images: (1) the correct detection rate: the number of correct detections divided by the total number of joints and (2) the distance error: the Euclidian distance (in pixels) of the detected center of the target joint from the GT coordinates. Since the X-ray images and hands have various scales and sizes, the X-ray images were first resized so that all images’ median lengths of the proximal phalanges matched. Next, bounding boxes were formed around the detected center points. The box sizes are 250 × 250 pixels for the PIP, IP, MCP, and CMC-M joints, 500 × 300 pixels for the radius, and 300 × 300 pixels for the others. For the correct detection rate, an orthopedic surgeon checked whether the bounding box correctly contained the target joint. If not, the box is treated as an error and discarded from the later experiment. For distance error, an orthopedic surgeon annotated the GT coordinate of the center of all target joints of both erosion and JSN for 50 X-ray images selected randomly from 206 test images. Then, the Euclidian distance of the detected target joint’s center from the GT coordinates was evaluated.

### Procedure for evaluation of classification models

The performance of the proposed four classification models was evaluated by using sensitivity, specificity, F-measure [38], and PR-AUC [39] with eightfold cross-validation. F-measure and PR-AUC are important indicators of classification model performance when there is a class imbalance [40, 41], as in this study. F-measure is the harmonic mean of sensitivity and precision, and PR-AUC is the curve of the area under the precision-recall curve, which is a plot of precision against sensitivity. F-measure and PR-AUC take values between 0 and 1 and become closer to 1 as performance improves.

Binary classification performance of the three orthopedic surgeons who were different from those who attached GT was also tested using the same 226 X-rays. These three orthopedic surgeons were not experts in RA. They evaluated each target joint for erosion and JSN as “intact” or “non-intact.”

## Results

### Joint detection results

Table 3 shows the correct detection rates for each target joint. The detection rates for each joint are as follows (intact, non-intact, total): for erosion, the PIP-IP joints (99.5%, 90.8%, 98.5%), the MCP joints (99.6%, 83.1%, 97.5%), the CMC-M (99.3%, 93.4%, 97.6%), the wrist joints (100.0%, 96.3%, 98.1%), and all joints (99.6%, 92.9%, 98.0%), and for JSN, the PIP joints (99.0%, 91.6%, 96.2%), the MCP joints (98.2%, 86.8%, 95.6%), the CMC joints (99.7%, 98.7%, 99.2%), the wrist joints (100.0%, 99.8%, 99.8%), and all joints (98.9%, 95.2%, 97.3%). On the whole, all the target joints were detected with high accuracy. Intact joints (SHS = 0) were detected correctly in most cases. Detection performance was generally good in the case of non-intact joints (SHS ≥ 1), although detection rates for the PIP-IP and MCP joints tended to be a little low for both erosion and JSN.

**Table 3:** Correct detection rates for test data

	Intact	Non-intact	Total
**Erosion**			
PIP-IP	916/921 (99.5%)	99/109 (90.8%)	1015/1030 (98.5%)
MCP	896/900 (99.6%)	108/130 (83.1%)	1004/1030 (97.5%)
CMC-M	289/291 (99.3%)	113/121 (93.4%)	402/412 (97.6%)
Wrist	393/393 (100.0%)	415/431 (96.3%)	806/824 (98.1%)
All joints	2494/2505 (99.6%)	735/791 (92.9%)	3229/3296 (98.0%)
**JSN**			
PIP	508/513 (99.0%)	285/311 (91.6%)	793/824 (96.2%)
MCP	782/796 (98.2%)	203/234 (86.8%)	985/1030 (95.6%)
CMC	304/305 (99.7%)	309/313 (98.7%)	613/618 (99.2%)
Wrist	197/197 (100.0%)	420/421 (99.8%)	617/618 (99.8%)
All joints	1791/1811 (98.9%)	1217/1279 (95.2%)	3008/3090 (97.3%)

Correct detection rates for intact (SHS = 0) joints, non-intact (SHS ≥ 1) joints, and the total for each target joint are shown. The numbers represent the correct detection/total cases (correct detection rates %). In erosion, “Wrist” represents the navicular, the lunate, the radius, and the ulna. In JSN, “Wrist” represents the multangular-navicular, the capitate-navicular-lunate, and the radiocarpal joint

*GT* ground-truth, *PIP* proximal interphalangeal, *IP* interphalangeal, *MCP* metacarpophalangeal, *CMC-M* carpometacarpal joint of the thumb and multangular, *JSN* joint space narrowing, *CMC* carpometacarpal

**Fig. 4:**
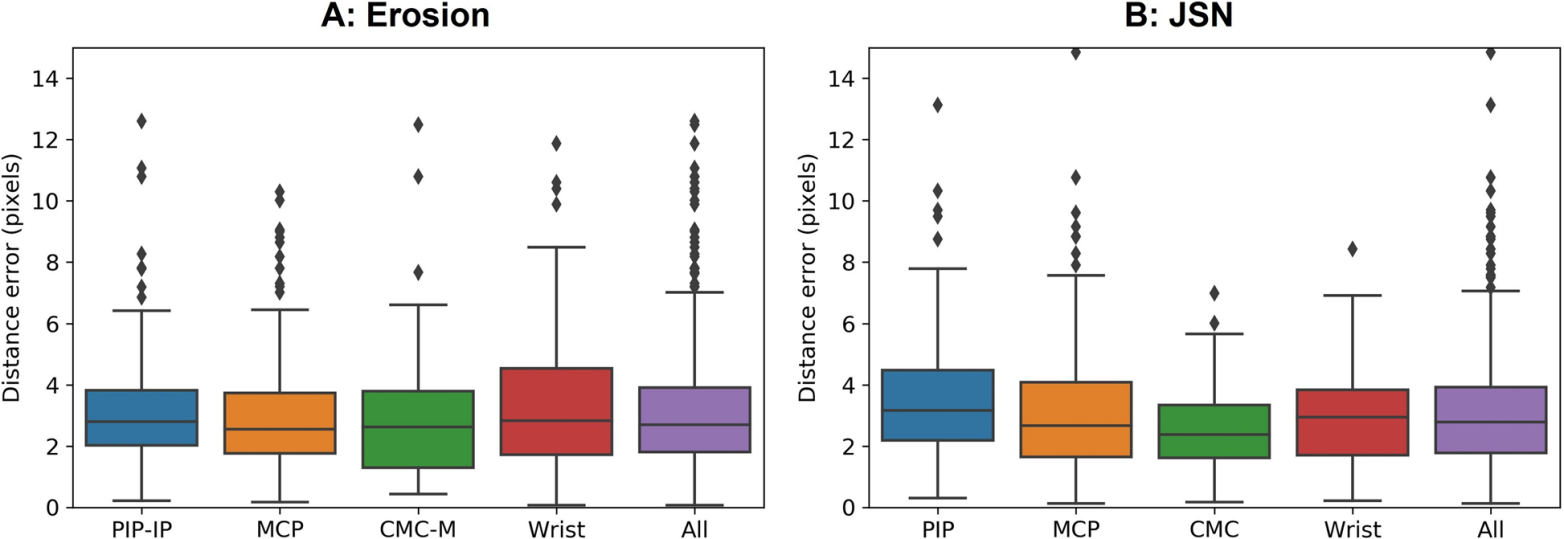
Boxplot of the distance error for each target joint of erosion and JSN. The distance error is defined as the Euclidian distance (pixels) of the detected center of target joints from the GT coordinate. For erosion, “Wrist” represents the navicular, the lunate, the radius, and the ulna; for JSN, “Wrist” represents the multangular-navicular, the capitate-navicular-lunate, and the radiocarpal joint. JSN, joint space narrowing; GT, ground-truth; PIP, proximal interphalangeal; IP, interphalangeal; MCP, metacarpophalangeal; CMC-M, carpometacarpal joint of the thumb and multangular; CMC, carpometacarpal

Figure 4 shows that the average distance errors were less than 6 pixels for all target joints for both erosion and JSN. The distance error for each joint are as follows (average ± SD pixels): for erosion, the PIP-IP joints (3.4 ± 3.5), the MCP joints (3.0 ± 2.6), the CMC-M (3.1 ± 2.9), the wrist joints (3.7 ± 4.1), and all joints (3.3 ± 3.4), and for JSN, the PIP joints (5.4 ± 9.4), the MCP joints (3.8 ± 6.5), the CMC joints (2.5 ± 1.2), the wrist joints (2.9 ± 1.5), and all joints (3.8 ± 6.3).

**Fig. 5:**
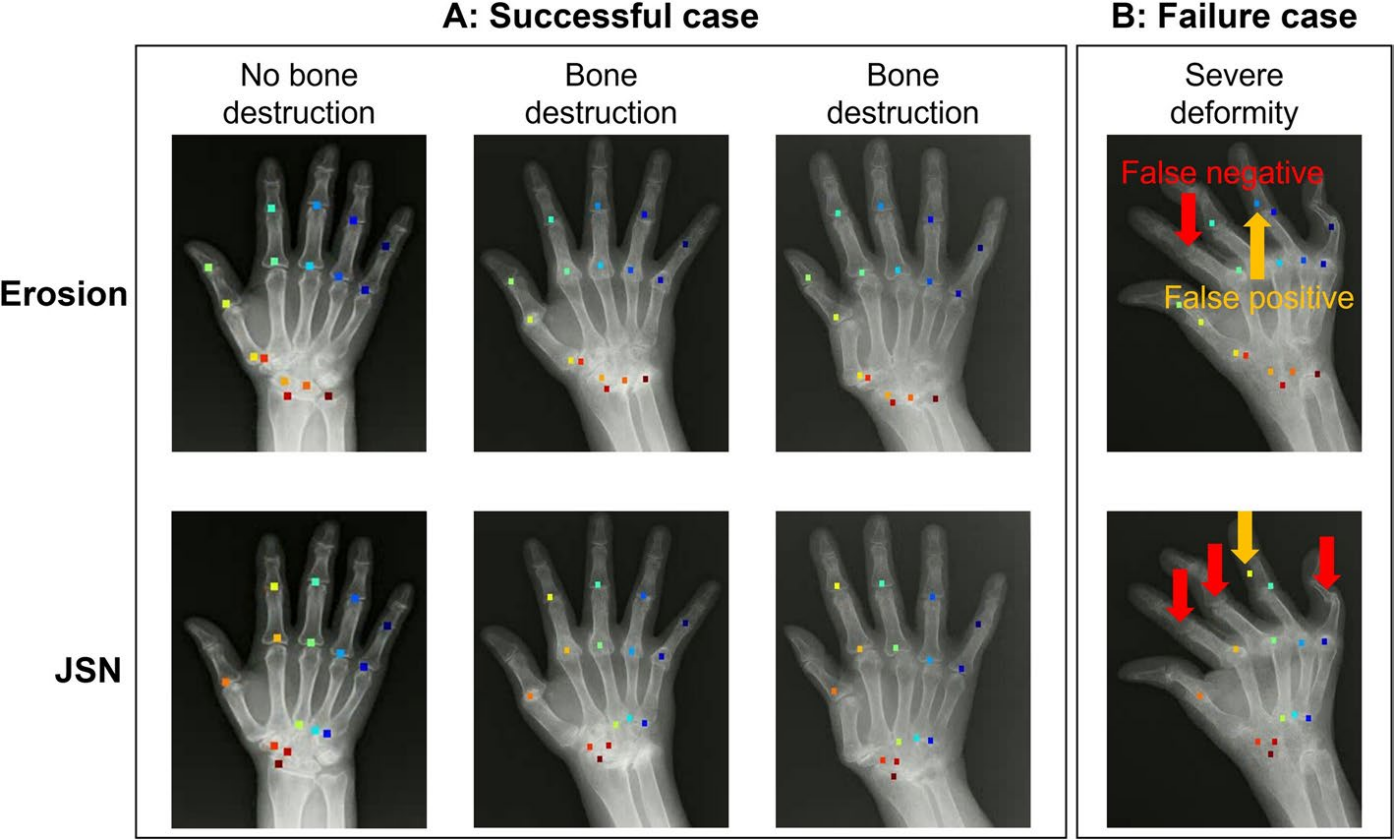
Detection results by DeepLabCut. Each point means the detected center point of the target joints, and each color corresponds to each joint. **A** DeepLabCut could detect the joints with high accuracy in cases with or without moderate bone destruction. **B** Detection failed in cases of severe bone destruction. The yellow arrow shows DeepLabCut detected the wrong area (false positive). The red arrow shows DeepLabCut could not detect the target joints (false negative)

Figure 5 shows examples of joint detection. In Fig. 5A, joints with no or moderate bone destruction were successfully detected. In Fig. 5B, in the case of severe bone destruction, false positives (yellow arrows) and false negatives (red arrows) for PIP and MCP joints were observed. On the contrary, the wrist joints could be detected accurately for both erosion and JSN, even when severe bone destruction was present.

### Results (classification)

**Fig. 6:**
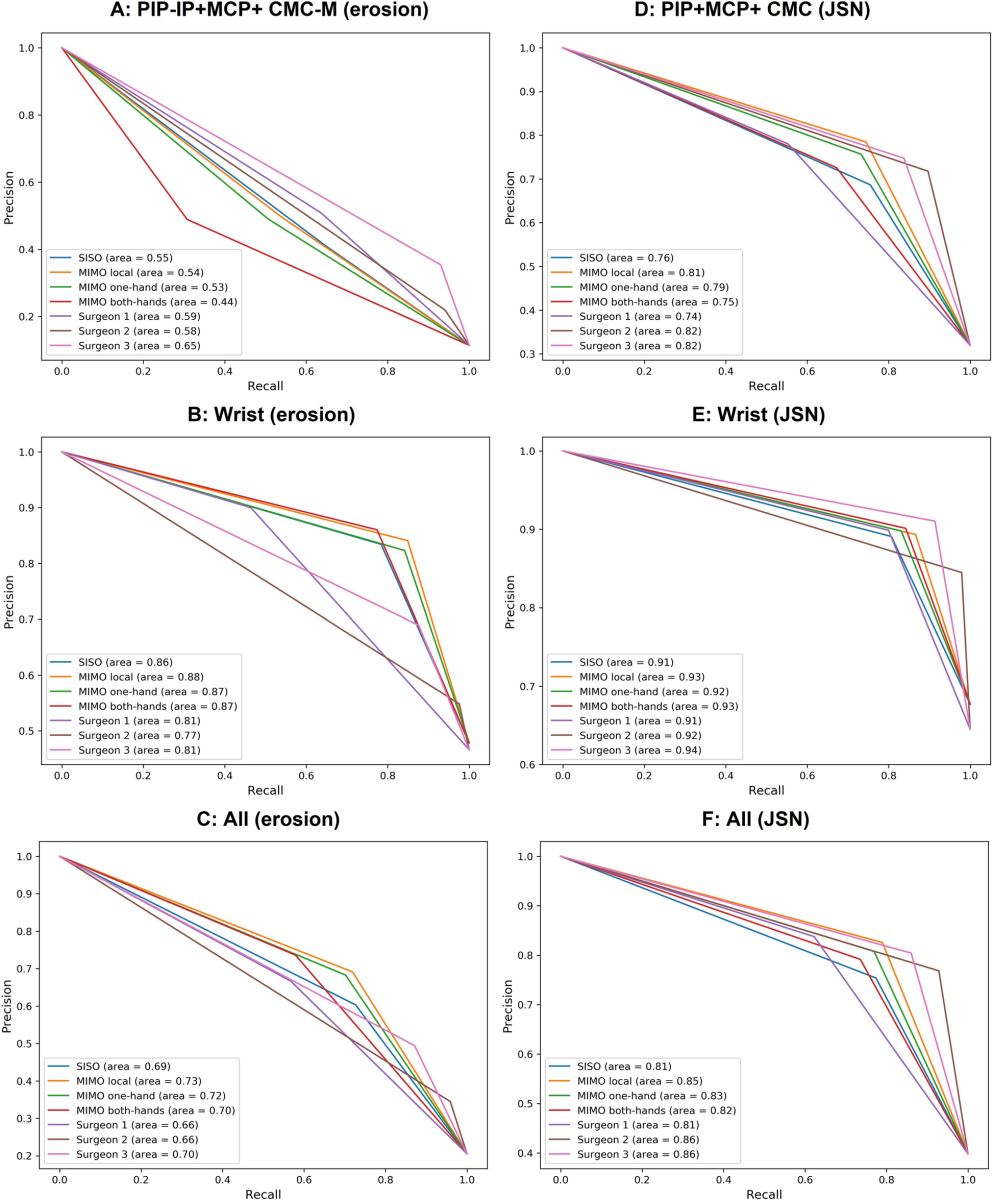
Precision-recall curve and area under the curve of each classification model and orthopedic surgeon for each target joint. For erosion, “Wrist” represents the navicular, the lunate, the radius, and the ulna; for JSN, “Wrist” represents the multangular-navicular, the capitate-navicular-lunate, and the radiocarpal joint. JSN, joint space narrowing; GT, ground-truth; PIP, proximal interphalangeal; IP, interphalangeal; MCP, metacarpophalangeal; CMC-M, carpometacarpal joint of the thumb and multangular; CMC, carpometacarpal; SISO, single-input single-output; MIMO, multiple-input multiple-output

Table 4 shows the binary classification performance of each classification model and each orthopedic surgeon for three groups (the wrist joints, the others, and all joints [the wrist joints and the others]). For all joints, in the case of both erosion and JSN, the MIMO local model and the MIMO one-hand model outperformed the SISO model in terms of F-measure and PR-AUC. Figure 6 shows the PR-curve. In addition, for all joints, the MIMO local model showed the best performance in terms of the following metrics: sensitivity of JSN (0.79), specificity of JSN (0.89), F-measure of erosion and JSN (0.70, 0.81), and PR-AUC of erosion and JSN (0.73, 0.85).

**Table 4:** Performance of binary classification

	SISO	MIMO local	MIMO one-hand	MIMO both-hands	Orthopedic surgeon 1	Orthopedic surgeon 2	Orthopedic surgeon 3	Average of surgeons
**PIP-IP+MCP+ CMC-M (erosion)**								
Sensitivity	0.65	0.54	0.51	0.31	0.64	**0.94**	0.93	0.84
Specificity	0.88	0.93	0.93	**0.96**	0.92	0.57	0.78	0.76
F-measure	0.50	0.52	0.50	0.38	**0.57**	0.36	0.51	0.48
PR-AUC	0.55	0.54	0.53	0.44	0.59	0.58	**0.65**	0.61
**Wrist (erosion)**								
Sensitivity	0.78	0.85	0.84	0.77	0.46	**0.98**	0.88	0.77
Specificity	0.86	0.85	0.83	0.88	**0.95**	0.30	0.65	0.64
F-measure	0.81	**0.84**	0.83	0.81	0.61	0.70	0.77	0.70
PR-AUC	0.86	**0.88**	0.87	0.87	0.81	0.77	0.81	0.80
**All joints (erosion)**								
Sensitivity	0.73	0.72	0.70	0.58	0.57	**0.96**	0.87	0.80
Specificity	0.88	0.92	0.92	**0.95**	0.93	0.53	0.77	0.74
F-measure	0.66	**0.70**	0.69	0.65	0.61	0.51	0.63	0.58
PR-AUC	0.69	**0.73**	0.72	0.70	0.66	0.66	0.70	0.67
**PIP+MCP+CMC (JSN)**								
Sensitivity	0.75	0.74	0.73	0.67	0.55	**0.90**	0.84	0.76
Specificity	0.84	0.90	0.89	0.88	**0.93**	0.83	0.87	0.88
F-measure	0.72	0.76	0.74	0.70	0.65	**0.80**	0.79	0.74
PR-AUC	0.76	0.81	0.79	0.75	0.74	**0.82**	0.82	0.79
**Wrist (JSN)**								
Sensitivity	0.81	0.87	0.83	0.84	0.80	**0.98**	0.91	0.90
Specificity	0.79	0.78	0.80	0.81	**0.84**	0.67	**0.84**	0.78
F-measure	0.85	0.88	0.86	0.87	0.85	0.91	**0.91**	0.89
PR-AUC	0.91	0.93	0.92	0.93	0.91	0.92	**0.94**	0.92
**All joints (JSN)**								
Sensitivity	0.77	0.79	0.77	0.74	0.62	**0.93**	0.86	0.80
Specificity	0.83	0.89	0.88	0.87	**0.92**	0.81	0.86	0.87
F-measure	0.76	0.81	0.79	0.76	0.71	**0.84**	0.83	0.80
PR-AUC	0.81	0.85	0.83	0.82	0.81	**0.86**	0.86	0.84

The performance of each classification model and orthopedic surgeons for erosion and JSN are shown. For erosion, “Wrist” represents the navicular, the lunate, the radius, and the ulna. For JSN, “Wrist” represents the multangular-navicular, the capitate-navicular-lunate, and the radiocarpal joint

*SISO* single-input single-output model, *MIMO* multiple-input multiple-output, *JSN* joint space narrowing, *PR-AUC* precision-recall area under the curve, *PIP* proximal interphalangeal, *IP* interphalangeal, *MCP* metacarpophalangeal, *CMC-M* carpometacarpal joint of thumb and multangular, *CMC* carpometacarpal

Furthermore, as for the F-measure and PR-AUC in the case of all joints, the MIMO local model showed better erosion classification performance than the best orthopedic surgeon. For JSN, this model was still better than the average of the orthopedic surgeons. For all joints, F-measure and PR-AUC were as follows (MIMO local model, average of the orthopedic surgeons, best of the orthopedic surgeons): F-measure (0.70, 0.58, 0.63) and PR-AUC (0.73, 0.67, 0.70) for erosion and F-measure (0.81, 0.80, 0.84) and PR-AUC (0.85, 0.84, 0.86) for JSN.

**Fig. 7:**
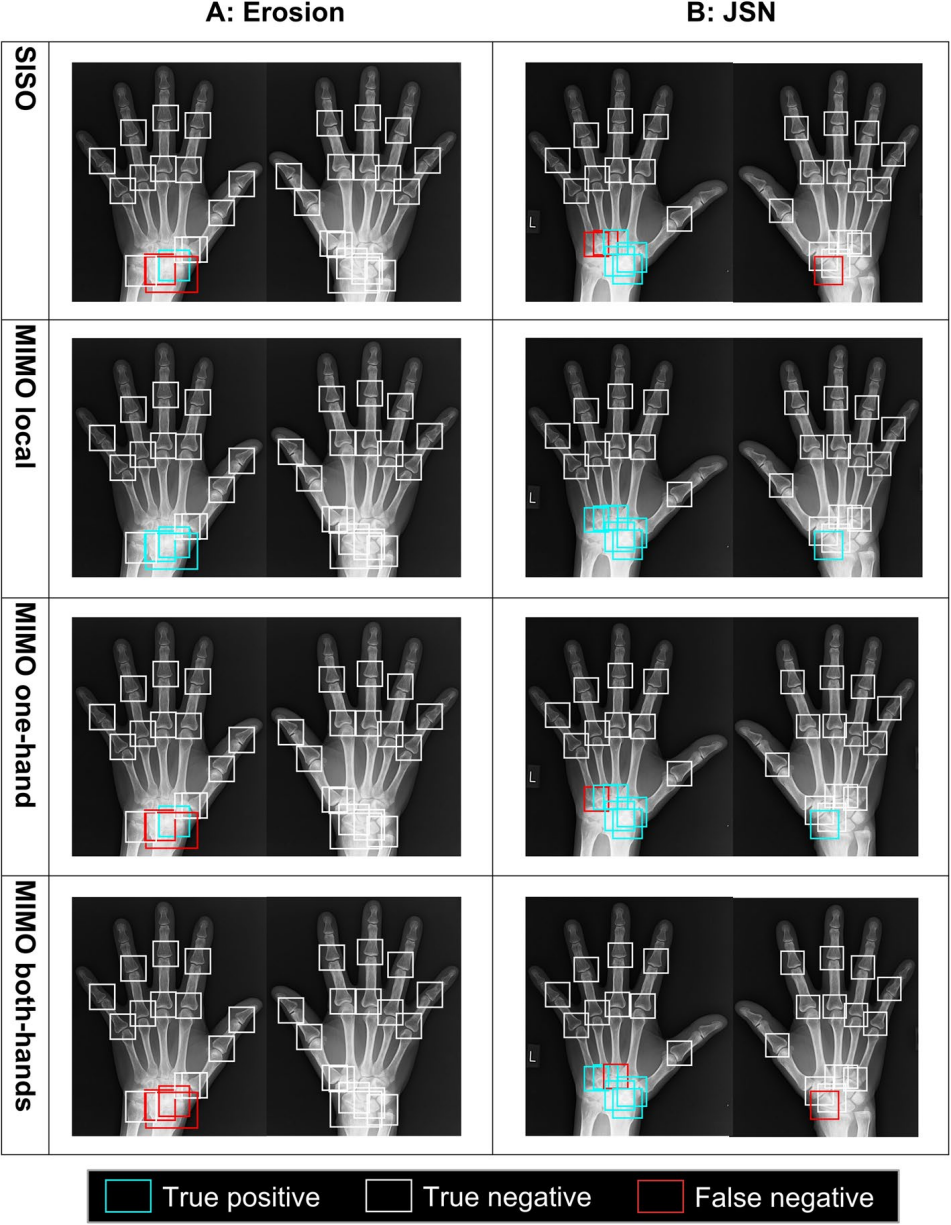
Visualization results of prediction by each classification model of target joints in X-ray images. Each bounding box represents the detected target joints. The color of the bounding box indicates how the classification results compare to ground-truth (GT). Blue indicates GT is non-intact, and prediction is non-intact (true positive). White indicates GT is intact, and prediction is intact (true negative). Red indicates GT is non-intact, and prediction is intact (false negative). **A** The results for erosion and **B** for JSN. GT, ground-truth; JSN, joint space narrowing; SISO, single-input single-output; MIMO, multiple-input multiple-output

Figure 7 shows examples of the visualization results of prediction by each classification model. In Fig. 7A, the MIMO local model correctly classified the left hand’s navicular, lunate, and radius as non-intact, although the other models misrecognized them. For JSN, the MIMO local model correctly classified the right hand’s radiocarpal joint and the left hand’s CMC joints as non-intact, although the other models misrecognized them (Fig. 7B).

## Discussion

The joint-detection performance of the proposed automatic-bone-destruction-evaluation system was very high for all the target joints. As for the binary classification, the MIMO local model, which could compare the same contralateral joint, showed the best performance for erosion and JSN regarding the F-measure and PR-AUC among the four classification models. In addition, the performance of this model for erosion was higher than that of the orthopedic surgeons. The MIMO local model’s classification performance of JSN was slightly lower than that of the best orthopedic surgeon but was slightly better than the average of the orthopedic surgeons. Since erosion is more critical than JSN for early diagnosis of RA [42], it has a clinical significance that the MIMO local model outperformed the orthopedic surgeon’s performance on erosion.

As for automatic detection, all the target joints could be detected very accurately, as shown in Table 3. Past reports [12, 14] focused on changes in brightness values for automatic detection. These methods are effective when the joint space is well defined, such as in the PIP-IP and MCP joints, but it is ineffective in the case of anatomically complex structures such as the navicular and lunate [12, 14]. DeepLabCut accurately detected all the target joints by learning the anatomical position and relationship between each target joint. Furthermore, DeepLabCut’s detection rates were better than previous reports [12, 14], although a direct comparison is difficult under different datasets. Hirano et al. [12] reported detection rates of 96.0% for the PIP-IP joints and 94.0% for the MCP joints. Morita et al. reported a detection rate of 91.8% for the 28 joints of the PIP joints, distal IP joints, and MCP joints. DeepLabCut’s detection rates were 98.5% for the PIP-IP joints, 97.5% for the MCP joints, and 98.0% for the PIP-IP and MCP joints (Table 3). DeepLabCut showed higher detection performance of the PIP-IP and MCP joints than the previous reports [12, 14].

When ulnar drift occurs in the PIP and MCP joints (Fig. 5B), detection by the proposed system tends to fail. This tendency can be explained by a change in the anatomical positional relationship between the proximal phalanges and the metacarpal bone. In contrast, the wrist joints could be appropriately detected, even though RA had progressed, because they had less anatomical deviation than the finger joints [43].

As for binary classification, the MIMO local model achieved the best performance among the four classification models. Comparing the same contralateral joint was more effective than comparing the same joint group. Previous studies [16, 42] reported that comparing contralateral joints improves the performance of reading X-ray images and many rheumatologists have used this comparison technique to diagnose bone destruction. Although the MIMO both-hands model also compares joints of both hands, its performance was not better than that of the MIMO local model and the MIMO one-hand model. We consider that increasing the number of input joints requires a combinatorial increase of training data, so it makes sufficient training difficult. Thus, selecting effective input images for a classification model is necessary.

In the non-intact cases, the MIMO local model and the MIMO one-hand model were effective in the wrist joints for both erosion and JSN. Bone destruction of the wrist joints progresses more symmetrically than the finger joints [16, 44]; therefore, the MIMO local model was suitable for the wrist joints. The MIMO one-hand model was also effective in the wrist joints because the group of the wrist joints had a similar progression of bone destruction.

**Fig. 8:**
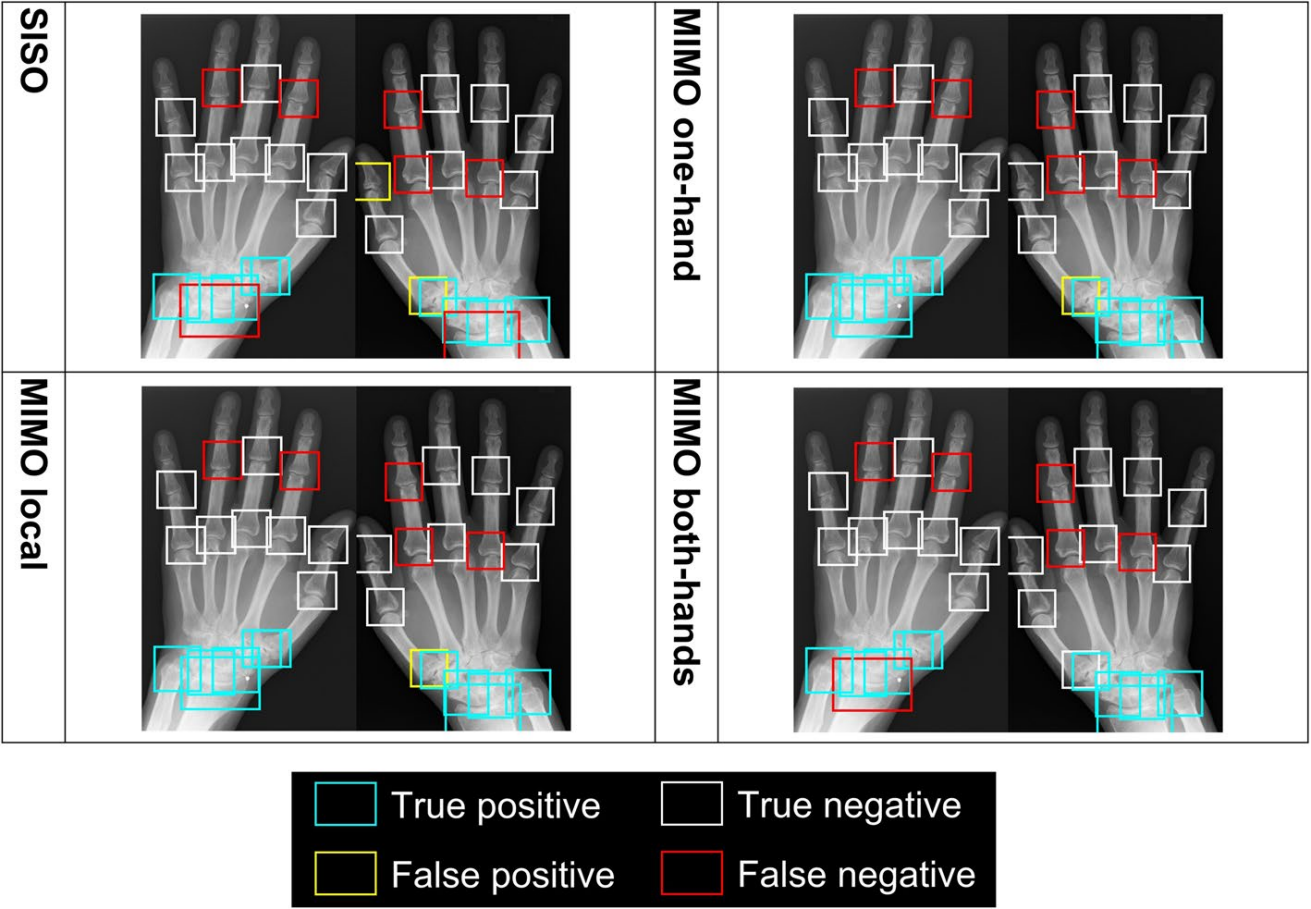
The difficult case of erosion for all four classification models. Each bounding box represents the detected target joints. The color of the bounding box indicates how the classification results compare to ground-truth (GT). Blue indicates GT is non-intact, and prediction is non-intact (true positive). White indicates GT is intact, and prediction is intact (true negative). Yellow indicates GT is intact, and prediction is non-intact (false positive). Red indicates GT is non-intact, and prediction is intact (false negative). GT, ground-truth; SISO, single-input single-output; MIMO, multiple-input multiple-output

In the case of mild bone destruction in erosion, which is important for early diagnosis [42], the four classification models showed relatively good performance for the wrist joints but not for the PIP-IP, MCP, and CMC-M joints. Figure 8 shows a difficult case for all four classification models. In the wrist joints of Fig. 8, 4 classification models could classify as non-intact correctly for almost all. However, all classification models misclassified the PIP and MCP joints as intact. These misclassifications could be explained by the mild degree of bone destruction. It is even difficult for rheumatologists to accurately discriminate between intact bone and mild bone destruction [45]. The classification models may therefore have difficulty in learning features that discriminate between intact bone and mild bone destruction. A possible reason for the higher accuracy in the wrist joints is that the wrist joints had less class imbalance. For the PIP-IP, MCP, and CMC-M joints, prediction performance may be improved by increasing the number of non-intact cases.

The best-performing classification model was the MIMO local model; in particular, it shows better classification performance regarding F-measure and PR-AUC than the orthopedic surgeons for all joints in the case of erosion. As for JSN, general orthopedic surgeons are more familiar with evaluating it than erosion because they treat several diseases (such as osteoarthritis) for which evaluating JSN is meaningful. Thus, their performance for JSN was better than erosion. Despite such a situation, the MIMO local model is slightly better than the average of the orthopedic surgeons for all joints in the case of JSN. Erosion is more important than JSN in diagnosing RA [42]. Therefore, it has a clinical significance that the MIMO local model performed better than the orthopedic surgeons in the case of erosion. We thus conclude that the MIMO local model has a higher classification ability than the orthopedic surgeons in the case of erosion.

The limitations of this study are that we could use a relatively small number of hand data. More data may improve classification performance in the case of mild bone destruction in finger joints. If we have feet data, its combination with hand data would be helpful, especially for early diagnosis of RA [46].

In future work, we aim to improve classification performance by incorporating time-series information into the classification models, which is helpful for SHS scoring [9, 47]. Using the same framework as the MIMO local model will make it possible to incorporate time-series information.

## Conclusions

In conclusion, the proposed automatic-bone-destruction-evaluation system was effective. As for automatic detection, all the target joints were detected with high accuracy. As for automatic binary classification, the proposed classification method, which could compare the same contralateral joint, showed good classification performance for both erosion and JSN. In addition, the classification performance by the proposed method was better than that of the three orthopedic surgeons for erosion.

## Supplementary Information

**Additional file 1: Table S1**. Final settings of hyperparameters (erosion). Each classification model’s final settings of hyperparameters for each target joint are shown. “Wrist” represents the navicular, the lunate, the radius, and the ulna. Note: FC, fully connected; PIP, proximal interphalangeal; IP, interphalangeal; MCP, metacarpophalangeal; CMC-M, carpometacarpal joint of the thumb and multangular.

**Additional file 2: Table S2**. Final settings of hyperparameters (JSN). Each classification model’s final settings of hyperparameters for each target joint are shown. "Wrist" represents the multangular-navicular, the capitate-navicular-lunate, and the radiocarpal joint. Note: JSN, joint space narrowing; FC, fully connected; PIP, proximal interphalangeal; MCP, metacarpophalangeal; CMC, carpometacarpal.

## Data Availability

The data that support the findings of this study are available from the corresponding author, KM, upon reasonable request.

## Abbreviations

AI: Artificial intelligence
CMC: Carpometacarpal
CMC-M: Carpometacarpal joint of the thumb and multangular
DNN: Deep neural networks
GT: Ground-truth
IP: Interphalangeal
JSN: Joint space narrowing
MCP: Metacarpophalangeal
MIMO: Multiple-input multiple-output
PIP: Proximal interphalangeal
RA: Rheumatoid arthritis
SHS: Modified Sharp/van der Heijde score
SISO: Single-input single-output
