# Supplementary material for: Deep learning-based automatic-bone-destruction-evaluation system using contextual information from other joints
Source: Arthritis Res Ther. 2022 Oct 3;24:227. doi: 10.1186/s13075-022-02914-7 (PMC9528108; doi:10.1186/s13075-022-02914-7)
Supplement: Supplementary file 1 — Additional file 1: Table S1. Final settings of hyperparameters (erosion). Each classification model’s final settings of hyperparameters for each target joint are shown. “Wrist” represents the navicular, the lunate, the radius, and the ulna. Note: FC, fully connected; PIP, proximal interphalangeal; IP, interphalangeal; MCP, metacarpophalangeal; CMC-M, carpometacarpal joint of the thumb and multangular. [file 13075_2022_2914_MOESM1_ESM.docx]

**Supplementary Information**

**Table S1.** Final settings of hyperparameters (erosion)

| **SISO (erosion)** | **FC layer** | **Initialization** | **Dropout** | **Batch size** |
| --- | --- | --- | --- | --- |
| PIP-IP | 1 | Random | - | 32 |
| MCP | 1 | He | - | 32 |
| CMC-M | 1 | He | + | 32 |
| Carpal bones | 1 | Random | + | 32 |
| Radius | 1 | He | + | 32 |
| Ulna | 1 | Random | - | 32 |
| **MIMO local**  **(erosion)** |  |  |  |  |
| PIP-IP | 1 | He | + | 32 |
| MCP | 1 | Random | + | 32 |
| CMC-M | 3 | Random | - | 32 |
| Carpal bones | 3 | He | - | 32 |
| Radius | 3 | He | - | 32 |
| Ulna | 3 | He | - | 32 |
| **MIMO one-hand**  **(erosion)** |  |  |  |  |
| PIP-IP | 3 | He | + | 32 |
| MCP | 1 | He | + | 32 |
| CMC-M | 3 | Random | - | 32 |
| Wrist | 3 | He | - | 64 |
| **MIMO both-hands**  **(erosion)** |  |  |  |  |
| PIP-IP | 3 | Random | + | 128 |
| MCP | 1 | Random | + | 32 |
| CMC-M | 3 | Random | - | 64 |
| Wrist | 3 | He | - | 64 |

Each classification model's final settings of hyperparameters for each target joint are shown.

"Wrist" represents the navicular, the lunate, the radius, and the ulna.

Note: FC, fully connected; PIP, proximal interphalangeal; IP, interphalangeal; MCP, metacarpophalangeal; CMC-M, carpometacarpal joint of the thumb and multangular.
