# Supplementary material for: Deep learning-based automatic-bone-destruction-evaluation system using contextual information from other joints
Source: Arthritis Res Ther. 2022 Oct 3;24:227. doi: 10.1186/s13075-022-02914-7 (PMC9528108; doi:10.1186/s13075-022-02914-7)
Supplement: Supplementary file 2 — Additional file 2: Table S2. Final settings of hyperparameters (JSN). Each classification model’s final settings of hyperparameters for each target joint are shown. "Wrist" represents the multangular-navicular, the capitate-navicular-lunate, and the radiocarpal joint. Note: JSN, joint space narrowing; FC, fully connected; PIP, proximal interphalangeal; MCP, metacarpophalangeal; CMC, carpometacarpal. [file 13075_2022_2914_MOESM2_ESM.docx]

**Table S2.** Final settings of hyperparameters (JSN)

| **SISO (JSN)** | **FC layer** | **Initialization** | **Dropout** | **Batch size** |
| --- | --- | --- | --- | --- |
| PIP | 1 | He | - | 32 |
| MCP | 1 | He | + | 32 |
| CMC | 1 | He | - | 32 |
| Midcarpal | 1 | Random | + | 32 |
| Radiocarpal | 1 | He | + | 32 |
| **MIMO local (JSN)** |  |  |  |  |
| PIP | 3 | Random | - | 64 |
| MCP | 1 | Random | + | 32 |
| CMC | 3 | He | - | 32 |
| Midcarpal | 3 | He | - | 64 |
| Radiocarpal | 3 | Random | - | 32 |
| **MIMO one-hand**  **(JSN)** |  |  |  |  |
| PIP | 3 | Random | - | 64 |
| MCP | 1 | Random | - | 32 |
| CMC | 3 | He | - | 32 |
| Wrist | 3 | He | - | 32 |
| **MIMO both-hands**  **(JSN)** |  |  |  |  |
| PIP | 3 | Random | - | 64 |
| MCP | 1 | Random | - | 32 |
| CMC | 3 | He | - | 32 |
| Wrist | 3 | He | - | 32 |

Each classification model's final settings of hyperparameters for each target joint are shown.

"Wrist" represents the multangular-navicular, the capitate-navicular-lunate, and the radiocarpal joint.

Note: JSN, joint space narrowing; FC, fully connected; PIP, proximal interphalangeal; MCP, metacarpophalangeal; CMC, carpometacar
